# Notes and Notices

**Published:** 1895-05

**Authors:** 


### NOTES AND NOTICES.

Dr. Charles Gatchell has again resumed the editorial pen of *The North American Journal of Homœopathy*. Dr. Gatchell's return will be warmly welcomed by the profession and his brother journalists.

The University of Michigan is now reorganizing the homœopathic department. We would respectfully suggest to the regents that they select for Dean Prof. L. D. Rogers, A. M., M. D., of Chicago. He is a good homœopathist, a well educated physician, and an author. He is editor of *The People's Health Journal* of Chicago, and Professor in one of the homœopathic colleges in that city.

The International Hahnemannian Association will hold its next annual meeting Wednesday, June 26th, 1895, at Watch Hill, Rhode Island. For all information apply to Howard Crutcher, M. D., 1102 Colonial Memorial Building, Chicago, Illinois.

The National Medical College.—The fifth regular course of lectures of the National Medical College will open April 2d, and continue six months. The full work of the College will be given, and also some additional advantages will be offered students during this summer semester.

This course will be divided into a spring term (April to June) and a summer term (July to September inclusive). Students can enter at the opening of either term, but must attend six full months to count time on a regular course. Teachers desiring to become physicians can here spend their vacations most profitably.

Special preparatory instruction will be given during this course in the Physical and Natural Sciences and in Latin to those students who may need it.

For annual announcement and catalogue giving full information, address

Prof. A. G. Thome, A. M., M. D., Registrar,
239 Lincoln Avenue, Chicago, Ill.

A Map of the World has been issued by the Rio Chemical Co., 401 North Main Street, St. Louis, Mo. It is arranged on Mercator's projection and is corrected to the present time. Its size is 27 inches length by 27 inches wide and is arranged to be hung up in the office. A copy will be sent free to every physician in America and Europe. It is a fine piece of work and well worthy of a place in the office.

